# Toxicological Findings of Self-Poisoning Suicidal Deaths: A Systematic Review by Countries

**DOI:** 10.3390/toxics10110654

**Published:** 2022-10-29

**Authors:** Giuseppe Davide Albano, Ginevra Malta, Corinne La Spina, Arianna Rifiorito, Valeria Provenzano, Valentina Triolo, Fabio Vaiano, Elisabetta Bertol, Stefania Zerbo, Antonina Argo

**Affiliations:** 1 PROMISE Department, University of Palermo, Piazza Marina 61, 90133 Palermo, Italy; 2 Department of Health Sciences, University of Florence, 50121 Florence, Italy

**Keywords:** poisoning, suicide, forensic toxicology, autopsy, systematic review

## Abstract

The use of illicit and non-illicit substances is widespread in suicides. The toxicological data may help in understanding the mechanism of death. This systematic review aimed to analyze autopsies related to suicides by consuming poison, focusing on the correlation between substance use and the country of origin to create an alarm bell to indicate that suicide maybe attempted and prevent it. The systematic review was conducted according to the PRISMA guidelines, with the primary objective of identifying autopsies conducted in cases of suicide by consuming poison in specific geographic areas. Significant differences in substances were observed between low-income and Western countries that confirm previous literature data. In rural areas and Asian countries, most suicides by consuming poison involve the use of pesticides, such as organophosphates and carbamates. In Western countries, illicit drugs and medically prescribed drugs are the leading cause of suicide by self-poisoning. Future research should shed light on the correlation between social, medical, and demographic characteristics and the autopsy findings in suicides by self-poisoning to highlight the risk factors and implement tailored prevention programs worldwide. Performing a complete autopsy on a suspected suicide by self-poisoning could be essential in supporting worldwide public health measures and policy makers. Therefore, complete autopsies in such cases must be vigorously promoted.

## 1. Introduction

Poisoning can result from exposure to drugs, chemicals, or toxins. Poisoning is a relevant public health issue responsible for a considerable number of cases of morbidity and mortality worldwide, especially in low- and middle-income countries [1]. According to the World Health Organization (WHO), in 2000, unintentional poisoning led to around 350,000 deaths [2]. Meanwhile, around 250,000 deaths resulted from intentional ingestion [3]. One report found that in 2012,about 1,930,000 people died worldwide from unintentional poisoning, of which 84% died in low- and middle-income countries [4]. A2019 WHO report indicates a worldwide decrease in the global age-standardized suicide rate [5,6].

To shed light on the cause and manner of the death, toxicological analysis is crucial in the forensic context, combined with a historical anamnesis, scene evidence, psychological autopsy, and autopsy. The investigation of deaths by poisoning requires a standardized methodology, the cooperation of experts in multiple forensic sciences, cross-examination, and a cross-talk between laboratory toxicologists and pathologists [7,8]. However, toxicological forensic diagnosis is extremely varied, with significant differences in laboratory capacities and technical developments across countries [9,10].

Socio-cultural background and legislation influence rates and methods of suicide. Therefore, significant differences can be observed between countries [6,11]. Intentional self-poisoning is one of the most frequently used methods of committing suicide, along with using firearms and committing suicide by hanging [12]. Self-poisoning accounts for 25% of total suicides in the United Kingdom and is the most frequent method of committing suicide among the younger population in the U.S. [13,14]. Rates of suicide by consuming poison are higher in developing countries, especially suicide by consuming a pesticide. In high-income countries, drugs used in attempts to commit suicide include psychotropic drugs, analgesics, antihistamines, antidepressants, psychoactive drugs, and sedative-hypnotics [15].

Research on methods of committing suicide and the risk factors involved in suicide by consuming poison is still inadequate. Moreover, literature studies are often not homogeneous, leading to inconsistent and non-representative socio-demographic and public health analyses of such phenomena. To raise awareness in the community and for future political and health strategies, this systematic review aimed to analyze autopsies related to suicide by self-poisoning, focusing on toxicological findings, the keys to the toxicological diagnosis, and the differences among countries worldwide.

## 2. Materials and Methods

### 2.1. Protocol

A systematic literature search was conducted by two authors (G.M. and G.D.A.) independently for studies published between January 2000 and May 2022. The protocol for this study was designed following the Preferred Reporting Items for Systematic Reviews and Meta-Analyses (PRISMA) guidelines [16] using the methodology described in the Cochrane Collaboration Handbook on Systematic Reviews of Health Promotion and Public Health Program [17].

### 2.2. Data Sources and Search Strategy

The records were identified using different search engines (PubMed and SCOPUS). For the search, MeSH terms and free text words were combined through Boolean operators as follows: Suicide AND ((Autopsy) OR (Forensic toxicology) OR (Forensic)) AND ((Drugs) OR (Poisoning)). The research was completed in May 2022.

### 2.3. Inclusion and Exclusion Criteria

Studies of autopsy series in a specific country with data regarding suicidal deaths by self-poisoning were included. The inclusion criteria for the studies were as follows:

- The article was in English.
- The article was original.
- The study involved human autopsies with data regarding suicide by consuming a variety of substances that can act as poison.
- The study had at least 20 patients.

We excluded (1) reviews, (2) case reports, (3) posters, abstracts, and communications at conferences, (4) articles not in English, (5) in vivo and in vitro studies, (6) articles not concerning a specific country, and (7) articles that evaluated self-poisoning by a specific substance.

### 2.4. Study Selection and Data Collection Process

**Figure 1:**
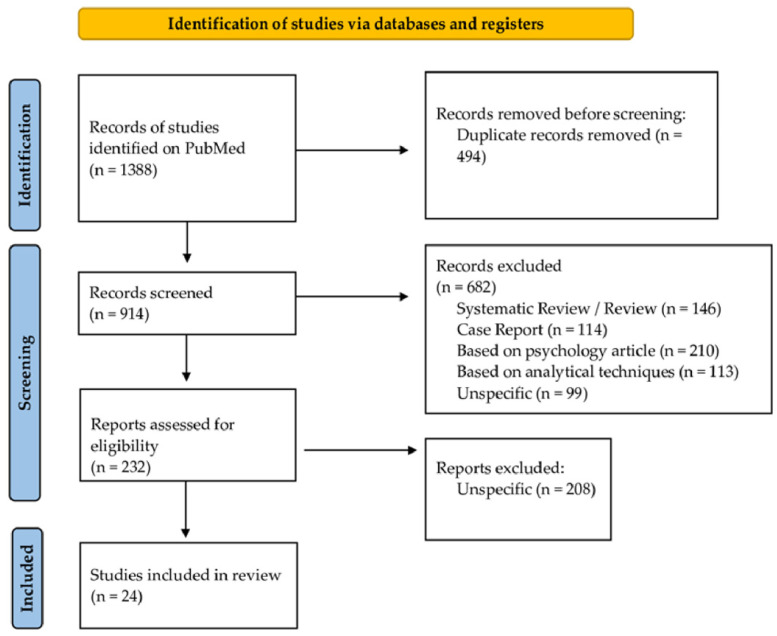
Flow diagram illustrating the search strategy and included and excluded studies in this systematic review.

Initially, articles were selected on the basis of title and abstract. Subsequently, a full-text evaluation of the selected studies was carried out. On the basis of the literature search, 1338 studies were identified. To begin with, 494 duplicates were removed, and 914 records were screened. Then, 682 records were excluded as per the exclusion criteria. After full-text evaluation, another 208 records were excluded because they were unspecific. The quality of each study was evaluated independently by G.M. and G.D.A. If there was a conflict of opinions regarding the articles, they were submitted to A.A. Finally, 24 articles were included in the current review. A detailed flow chart of the selection process is provided in Figure 1.

For each study, three authors (C.L.S., A.R. and V.P.) extracted the following data using a pre-designed data extraction Excel sheet. Study characteristics (name of the first author, year of publication, name of the country where the study was performed, and study design), sample characteristics (number of autopsies, manner of death, number of suicides, number of suicides by self-poisoning, and anamneses of patients), and methodology and toxicological findings (toxicological analysis technique, main toxicological findings, and types of drugs detected) were collected when present.

## 3. Results

All the selected studies are summarized in Table 1.

### 3.1. Characteristics of Articles Included in the Systematic Review

The search strategy identified 24 descriptive studies for inclusion in this review. All articles focused on autopsies with data regarding suicide by poisoning. The selected studies were geographically distributed as follows: 9 in Asia (3 in India (of which 1 was in South India), 3 in China, 1 in Iran, 1 in Pakistan, and 1 in Turkey), 7 in Northern Europe (4 in Sweden, 2 in Norway, and 1 in Finland), 4 in Central and Southern Europe (1 in France, 1 in Greece, 1 in Spain, and 1 in Portugal), 2 in the USA, 1 in Africa (Tunisia), and 1 in Central America (Jamaica). Of the articles, 92% were retrospective studies. In all, there were 31,252 cases of poisoning, of which14,276 cases involved suicide by poisoning. There were 6844 cases of accidental death by poisoning. About 42% (10/24) of the included articles were autopsy series on suicide, about 29% (7/24) were autopsy series with no specific manner of death identified, about 21% of the articles (5/24) focused on autopsies in poisoning cases, and in 8% of the studies (2/24) only suicide by self-poisoning autopsy series were examined. The mean age was specified in 13 articles. The predominant gender in the included studies was male (males: 18,028/25,195, or 71.5%; females: 7167/25,195, or 28.4%). In 7 articles, the distribution of gender was not specified. In the suicide autopsy series, consuming poison, hanging, and using firearms were the most common causes of death. In most articles, data on social and medical history were not detected. The most frequent methodologies for the toxicological analysis were GC-MS, GC, and LC-MS (performed in 5 studies). In 15 of the studies, the analytical technique performed was not specified. Pesticides were the most frequent substance used for suicide by self-poisoning in Asia and low-income countries. In Europe and Western countries, a lower pesticide incidence was observed, with higher rates of illicit drugs (especially opioids) and medically prescribed drugs (especially benzodiazepines, antidepressants, and neuroleptics) for suicide purposes.

### 3.2. Analysis of Autopsies Related to Suicide by Poisoning

#### 3.2.1. Africa

From 2005 to 2015, Gharbauoi et al. [25] conducted a descriptive cross-sectional study in Tunisia on a sample of 204 self-poisoning suicides examined across 10 years (2004–2014). The mean age of the victims was 56 years, with an M:F ratio of 0.6:1. In 22.1% of the cases, the victims had made previous suicide attempts. A history of psychiatric disorders was reported in 39.2% of the cases. Toxicological examinations revealed death by medical drugs (mainly psychotropics and cardiotropics) in 52.5% of the cases, pesticides in 42.6% of the cases, caustics in 4.4% of the cases, and butane gas in 0.5% of the cases. Fatal self-poisoning occurred more frequently in young, single, and unemployed women.

#### 3.2.2. Asia

In China, a retrospective study conducted on 299 autopsy cases by Pan et al. [23] showed that 13% (*n* = 39) of the deaths were poisoning cases, of which 12.8% (*n* = 5) were suicides. The remaining cases were attributed to accidental intake (69.2%), homicidal intentions (7.7%), and indeterminable causes (10.3%). Toxicological analyses were conducted by various methods, mainly GC-MS, on biological matrices such as urine, blood, hair, and liver. Mostly, the substances involved were drugs, volatile compounds, pesticides, and drugs of abuse.

In a retrospective study of Wang et al. [24] conducted on 1968 autopsies, 140 cases of death by poisoning (7.11%) were identified, 27.8% of which were suicides. Toxicological examinations of organs, blood, urine, and gastric contents reported medical drugs in 15.71% of the cases (22 cases, 11 hypnotics, antipsychotics, and 6 antiarrhythmic drugs).

Xiao et al. [22] studied 782 poison-related autopsy cases, 38.4% of which involved suicides by subjects with a mean age of 37 years, with a slight predominance of males over females. Toxicological investigations were conducted on blood, urine, and gastric content samples. They revealed the use of pesticides in 40% of the cases, toxic gases in 32% of the cases, drugs of abuse in 8.6% of the cases, and drugs and botanical derivatives in the rest.

The retrospective study by Goswami et al. [20] examined autopsies of suspected deaths by poisoning. Of the 584 cases, 41.8% (244) were of poisoning, of which, 86% (210) were cases of suicide. The remaining cases were of accidental poisoning and involved mainly adults. Most suicides by consuming poison involved the 21–30 age group (36.2%). Most poisoning cases occurred in rural areas (154, 63.1%). The most frequently found substance in toxicological analyses, carried out using GC-SM methods, were organophosphates (pesticides, 61.9%), followed by insecticides such as carbamates (18.4%) and other organic derivatives.

Another study [36], conducted retrospectively on 588 autopsy cases related to suicides, revealed that 30.6% of the cases were of suicide by the intentional consumption of organophosphates and local plants, followed by other compounds in agricultural use. The leading cause of suicide was hanging (32.6%). The mean age was about 25 years, with no particular gender difference. Most of the subjects were Hindu. A positive history of suicide attempts was present in 14% of the cases; meanwhile, in 22% of the cases, there was a history alcohol intake.

In South India, a retrospective study [18] of 10 years of autopsy records of suicide by poisoning confirmed the previous data (i.e., the use of mainly agricultural and industrial substances for suicidal purposes) but also analyzed further factors facilitating suicide, such as family problems (30.2%), chronic illnesses (28.2%), financial problems (11%), psychiatric problems (9.7%), and others.

A retrospective study conducted in Iran [26] examined 1667 autopsies and found that suicide by poisoning accounted for 45.8% of the sample. Toxicological examinations found that pesticides were involved in 84.2% of the cases, followed by opiates (6.8%), methamphetamines (2.74%), ethanol (1.3%), strychnine (1.3%), and minor drugs. The highest numbers of suicide by consuming poison were observed in the age group of 21–30yearsand in the unemployed category.

A retrospective study conducted by Azmak et al. [29] in Turkey on autopsies in suicide cases that occurred between 1984 and 2004 (137 cases in all) observed that the subjects had a mean age of 62.5 years, with an M:F ratio of 3:1. The primary mechanism of suicide was by hanging (40.1%), followed by the use of firearms (21.1%) and consumption of poison (19.7%). Toxicological investigations revealed that victims who had consumed poison had overdosed on single drugs or a mix of drugs in 51.8% of the cases, pesticides in 37% of the cases, acids in 7.4% of the cases, and methanol in 0.7% of the cases.

A retrospective study [42] in Pakistan analyzed the socio-demographic profiles of victims of poisoning where autopsy had been carried out. Out of the 300 cases selected for toxicological reports, 94 were suicides carried out mainly by taking insecticides, with an M:F ratio of 2:1 and a prevalence in the 21 to 40 age group. The motivations for suicide were primarily traced back to financial problems, failure in love, and health problems.

#### 3.2.3. Central America

A 20-year retrospective study [30] conducted in Jamaica on poisoning-related deaths revealed that 63.6% of these were suicides, committed by taking insecticides (41%), herbicides (paraquat 27%), drugs (48.9%), and cocaine (9%).

#### 3.2.4. Central and Southern Europe

Franchi et al. [28] conducted a comparative study between suicide autopsy cases in 2002 and 2012 in France. Of the486 suicide autopsies, 19.5% were related to poisoning; most suicides were by hanging (30.86%). The mean age of the subjects was 42 years, with a predominance of males (M:F ratio of 1.85). Psychiatric problems were reported in 62.5% of the cases. Toxicological blood tests showed that benzodiazepines were present in 42.38% of the cases, antidepressants in 31.68% of the cases, and neuroleptics and mood stabilizers in others.

Collados-Ros et al. [19] conducted a retrospective study in Spain from 2013 to 2018 on 355 suicide autopsy cases, of which 13% (*n* = 46) were suicides by poisoning. Toxicological investigations revealed the presence of drugs of abuse, pesticides, lorazepam, and carbon monoxide.

A Portuguese retrospective study [32] on autopsies of 304 subjects found that 97 cases (31.9%) were of suicide by consuming poison, while 119 (39.1%) were of suicide by hanging. The mean age of the subjects was 40 years, with an M:F ratio of 2:1. The main substances detected in the drug tests were alcohol, drugs, drugs of abuse, and pesticides. This study shows that nowadays, hanging is the leading method of committing suicide, although, over the years, poisoning has been the primary method. A study of 1681 autopsies performed in Greece [33] from 1998 to 2010 found 126 (7.49%) cases of poison-related deaths; the number of suicides was 257 (15.2%), and of these, 20 (16%) were suicides by poisoning. GC was used for toxicological investigations on blood and urine and revealed that there was drug abuse in 60%of the cases, and carbon monoxide was involved in 19.8% of the cases; less than 10% of the self-poisoning agents were pesticides, acids, and drugs. The remaining cases were classified as accidental intake. The ingestion of pesticides was the third cause of deaths by poisoning and was used mostly in rural areas for suicide.

#### 3.2.5. Northern Europe

In Sweden, four retrospective studies with consistent autopsy series were included in the review.

In the study by Jones et al. [27] covering 1993–2010, 13,963 poisoning-related autopsies were examined, of which 4843 were suicides (34.68%). Toxicological analyses revealed that ethanol was involved in 55% of the cases, paracetamol in 21% of the cases, diazepam in 19% of the cases, morphine in a similar percentage of cases, and minor antidepressants in a few cases.

Holmgren et al. [35] analyzed a case history of autopsies in suicides from 1998 to 2007 and found that 2462 (22%) of the suicides had been committed by consuming poison. Toxicological tests were conducted on urine, blood, and vitreous humor and found that the main substances used were paracetamol, antidepressants, and antipsychotics. Sedatives and opioids were also present in a smaller percentage.

Jonsson et al. [39] studied the autopsies of 60,313 subjects from 1992 to 2002 and found that 6966 (11.5%) were poison-related deaths and 44.3% were suicides. The mean age of the subjects was about 50 years, with an M:F ratio >1. The main toxic substances in peripheral blood were dextropropoxyphene, amitriptyline, zolpidem, methadone, and other antidepressants.

The same authors [37] studied case histories from 2002 to 2003. Out of 743 toxicological autopsies, 28 (3.7%) indicated poison-related suicides and13% indicated suicides not poison-related. Toxicological analyses on blood, urine, and vitreous humor revealed that 70% of the cases had involved drugs of abuse (morphine and psychostimulants) and ethanol. Medical histories revealed psychiatric problems. No illicit drugs were detected in 52 cases (53.6%). Illicit drugs were detected in 44 cases (45.3%).

A study by Delaviris et al. [31] on a sample of 1603 autopsy studies conducted between 2000 and 2009 showed that the mean age of the victims was 49.5 years and 7.11% (*n* = 114) of the cases involved suicide by poisoning. Blood samples were processed by qualitative and quantitative screening tests and showed that the subjects had taken morphine in 61.4% of the cases, amphetamines in 19.29% of the cases, cannabis in 15.7% of the cases, methadone in 14.9% of the cases, cocaine and ecstasy in a few cases.

A study conducted between 2007 and 2009 by Frost et al. [34] in Norway estimated that toxicological investigations were performed in 361 of 365 autopsies. The mean age was 44.5, and there was no gender analysis. Of the total sample, 2.4% involved suicide by poisoning. The collected matrices, blood, and urine were analyzed by GC, LC–MS and revealed the presence of benzodiazepines, alcohol, opioids, and psychoactive drugs. Cocaine and THC were found among the drugs of abuse.

A study published in 2003 by Lahti et al. [40] in Finland analyzed 500 autopsies conducted in 1997 where the mechanism of death was related to poison intake. Of these, 325 (65%) were cases of suicides, 99 (19.8%) were cases of accidental death, and 76 (15.2%) were undetermined. On toxicological analysis, 30.9% of the subjects tested positive for benzodiazepines, 21.7% for alcohol, 16.3% for cocaine and methamphetamines, 9.2% for selective serotonin reuptake inhibitors, 8.3% for cannabinoids, 3.9% for non-steroidal anti-inflammatory drugs, 2.9% for opiates, and 1.8% for methadone.

#### 3.2.6. The USA

In the USA, an elective study by Cuchara et al. [21] of suicide cases where autopsy was carried out revealed that out of 394 cases, 71 (18%) were attributable to poison intake. The most frequently detected substances in the toxicological examination were benzodiazepines, opioids, antidepressants, and ethanol. However, the most used methods were use of firearms and suicide by hanging, with an apparent reduction in the use of other means. Of note, the mean age was heterogeneous at 53 years but distributed mainly between adolescents and adults, with a positive psychiatric history in 45.9% of the cases.

Shieldz et al. [38] conducted a retrospective study in Kentucky in the period 1993–2002 and analyzed 2864 autopsies related to cases of suicide where the average age of the victim was 59 years. Of the victims, 2340 were men and 524 were women (M:F ratio = 6:1). Of the sample, 283 cases (9.9%) were related to poisoning, 393 (13.7%) cases to hanging, and 1932 (67.4%) cases to the use of firearms. The matrices subjected to toxicological analyses were blood and urine. Tests revealed the use of antidepressants in 54.4% of the cases, benzodiazepines in 29.2% of the cases, opioids in 37.4% of the cases, cannabinoids in 13.3% of the cases, and cocaine and related metabolites in 6% of the cases.

## 4. Discussion

This systematic review analyzed the cases of suicide carried out by consuming licit or illicit substances. The analysis focused on evaluating the number of suicides the geographical areas where these occurred and the correlation between social, medical, and demographic characteristics of the victim and the toxicological findings of the autopsy.

The phenomenon of suicide is heterogeneous throughout the world. The gender and age distribution of subjects is also heterogeneous, although not all studies have highlighted these aspects [41].

**Figure 2:**
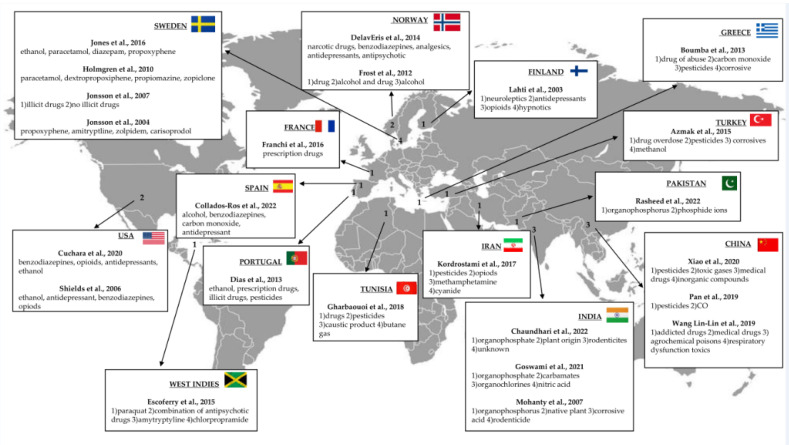
Representative figure illustrating the number of autopsies in cases of suicide by self-poisoning and the most frequent types of toxic substances used and their worldwide distribution, according to the articles included in this review [18,19,20,21,22,23,24,25,26,27,28,29,30,31,32,33,34,35,36,37,38,39,40,41].

The studies examined showed a clear majority of male subjects; however, there were no sufficient data on the type of suicide or the relationship between the kind of substance in case of self-poisoning and gender. This could be a basis for further investigation to shed the light on the emerging gender medicine [43]. In line with what has been observed, what is lacking in the study of most of the reviews carried out on suicide cases is an in-depth evaluation of clinical and medical history of the subjects. Significant differences in the substances used for suicide by self-poisoning were observed in the examined studies (Figure 2).

Concerning the type of substance used, it was observed that in low-income countries (India) or developed countries with significant agricultural areas (China), most suicides by poisoning occurred mainly through the use of pesticides such as organophosphates and carbamates, substances with muscarinic, nicotinic, and neurotoxic action [44]. This appears to be related to the easy availability of the means, as these are predominantly agricultural countries. In contrast, in Western or industrialized countries, use of illicit drugs (mainly opioids) and medicines (antidepressants, anxiolytics, and neuroleptics) for suicide appeared to be more frequent. More precisely, greater use of antidepressants than drug abuse has been observed over the years, probably due to their greater availability and the progressive increase in the incidence of psychiatric pathologies and stress-related disorders [45,46].

The prevention of suicide by self-poisoning is a relevant public health concern. Public health and worldwide authorities are responsible for addressing the increase in suicide rates, which is affecting all populations and our society [47]. Adequate legislation is mandatory to reduce this phenomenon. The role of healthcare providers (nurses, toxicologists, and public health professionals) is crucial in identifying the risk factors related to suicide and the toxicological issues of suicide by self-poisoning and implementing relevant policies. Psychiatric liaison, Child and Adolescent Mental Health Services, and primary care nurses must have in-depth knowledge and skills to conduct comprehensive assessments [13]. It is necessary to know the groups at high risk and adopt approaches that address the individual and societal factors that motivate suicide. In societies where basic needs of inhabitants, such as their emotional, financial, and religious needs, are addressed, suicide and its consequences rarely occur [48].

The WHO stated that self-poisoning using pesticides is one of the leading causes of suicide worldwide. Regulation and adequate legislation may considerably prevent this phenomenon.

In the past 20 years, a decrease in deaths due to pesticide poisoning has been observed (from around 260,000 a year to 160,000 a year). However, more than 150,000 people still die from intentional ingestion of pesticides, accounting for about 20% of the global burden of suicide. The decrease in deaths is believed to be due to tighter regulation and increased mechanization of agriculture, resulting in reduced numbers of agricultural workers. In many low-income countries in the Asia-Pacific region, suicide is the leading cause of death in early to middle adult life and pesticides account for around half to two-thirds of the suicides. These data correspond to those discussed in this systematic review and highlight that pesticide poisoning is still a global and relevant public health concern [12,14].

The types of substances involved in poisoning cases are continuously changing according to local environmental, cultural, and economic factors and differences in the management of poisonous substances in various countries [6].

According to the literature and the results of the present study, pesticides are the most used toxic products in suicides by consuming poison in developing countries with agricultural bases. In this regard, one of the selected studies reported that in Iran, one reason for this high drug use is the free availability of drugs without prescription from legitimate pharmacy channels and through non-medical sources [26].

Medical drugs are also a common means of fatal poisoning, mostly in developed countries, with increased hospitalizations for poisoning by prescription opioids, sedatives, and tranquilizers. The widespread use of these medical drugs, also due to medical prescriptions, has increased over time, as indicated by the selected study conducted in France. The role of the medical doctor is essential, and suicide by consuming medical drugs, especially in Western countries, highlights the necessity of training of the relevant doctors better [49,50,51].

Official statistics on suicides in 2016observed a decrease in the prevalence of self-poisoning for both males and females and a higher proportion of females using this suicide method (36.2% of females versus 18.3% of males).

With regard to suicides using toxic substances, the age of the victims was between 20 and 29 years in most cases of our study, which is comparable to previous reports. This is not a surprise. The young age of the victims correlates with their vulnerability and impulsiveness and their easy access to toxic drugs [52,53].

In this study, data from several geographic areas, such as Australia, Eastern Europe, Russia, and South America, are missing. Some manuscripts from these areas were initially assessed for eligibility and excluded after full-text reading because they did not adhere to the inclusion criteria. In Australia, according to a recent study [50], an increasing trend of deaths due to intentional consumption of poison was observed, with a significant increase in medical prescription drugs, especially opioids, as in other high-income countries. Moreover, Australian epidemiological data show a changing trend among different jurisdictions in terms of the manner of death due to poisoning. In most eastern European countries, according to the WHO mortality database on methods of suicide [54], poisoning is the third-leading cause of suicide death, after suicide by hanging and suicide by using firearms. Pharmaceutical agents are the most frequent victims of intentional suicide in this area. No autopsies related to suicide by poisoning were found in this area and other missing geographic regions according to our research and selection criteria. This could be an important indication for further research in the future. Although the young population is more likely to engage in risky behaviors, such as alcohol intake and illicit drug use, many studies do not evaluate the different mechanisms of suicide by age group and sex and it is challenging to establish the frequency of various suicide methods by different age and gender groups. However, the WHO highlights that some risk factors, such as harmful use of alcohol and substance use disorders, contribute to suicide ideation in all age groups [14,53]. According to one of the selected studies, the National Institute of Statistics in Spain argues that the number of suicides is higher in men than in women [55].

An American Psychiatric Association report highlighted the importance of several social and economic elements—single people may have the wrong social support network in their daily context. Depression and bad social conditions are related to a greater risk of suicide attempts, and family and social support is crucial to protection from suicide attempts. Isolation may trigger events related to vulnerability and lead to suicide. Both unemployment and low educational levels are significant risk factors for suicide attempts as they are associated with social drawbacks that may predispose an individual to suicidal behavior [49,56,57,58].

Depression was the most relevant risk factor associated with suicide. This disease, taken by its symptoms or other factors, usually involves physical and psychological distress; life terminality; social, economic, and cultural problems; losses; loneliness; and family conflicts. The robust association between suicide and depression demands that greater attention be paid to diagnosing this mental disorder, particularly in primary healthcare, and the interventions designed to treat depression are considered as essential to preventing suicide [12,58].

As suggested by Franchi et al. [28], new methods have been introduced to improve the general knowledge about suicide epidemiology. One of these is psychological autopsy, developed in the United States and a few other countries (primarily Nordic) and introduced in France, including Lyon, in the 1990s. By collecting circumstantial and socio-psychological data, it is possible to identify the factors and preclude suicide. All these elements indicate that suicide prevention in all countries should be rethought and reorganized. Furthermore, the role of the general practitioner should be strengthened in the care of suicidal patients because they are often on the front line. At the same time, psychiatrists are seldom directly solicited by these patients [56,58,59,60].

Research on suicide methods is still inadequate, especially in some geographic areas. Further and continuous research is needed in this field to investigate and update the critical risk and protective factors involved in suicide and to implement prevention programs. Considering the limited resources and the large population in many low-income countries, it could be helpful to adopt public health measures by referring to social, religious, and economic factors to prevent suicide in various countries [52,53].

This study has several limitations. The autopsy rate in deaths by suicide and poisoning is still low [21,22,23,24,25,26,27,28]. The data emerging from the studies included in the review only partially reflect the toxicological characteristics of the country where the study was conducted. The findings are limited, given that only articles published in the English language were included and that National data is often published locally. Furthermore, the selected studies lack significant anamnestic and socio-demographic data, which could be important in investigating the risk factors involved in suicide by consuming poison. Moreover, the data that correlated self-poisoning as the method of suicide lacked variables regarding essential issues such as illness-related factors and psychopathological issues, known to influence suicide risk. Moreover, in autopsy studies, it is hard to differentiate between medications taken for medical treatment as opposed to those taken only to commit suicide, especially in the case of medically prescribed drugs. Moreover, the coroner’s toxicology results focus on substances present at the moment of death. They do not always gather information about chronic exposure to substances, medically prescribed drugs, or the source of ingestion.

The data of the selected studies are coherent with literature data and highlight that the characteristics of suicide by consuming poison show changing trends according to social and demographic factors [52,61]. Autopsy and toxicological analysis in suicide deaths are crucial for assessing the cause and manner of death and shed light on death by self-poisoning. When performing an autopsy in the case of suspected suicides, it is crucial to analyze social factors and medical history to pinpoint a suicide by self-poisoning and provide helpful information for prevention and public health measures. In this regard, the results of the present study lack significant information about sociodemographic factors. There is strong evidence that a reduction in the availability of the types of drugs used in suicide by drug poisoning might indeed have a positive influence on reducing suicide rates.In this regard, autopsy, a complete toxicological panel, and complete evaluation of anamnesis and social history may have a key role when suicide by self-poisoning is suspected, to support prevention and public health measures.

## 5. Conclusions

The selected studies offer a snapshot of toxicological findings of autopsies in cases of suicide by self-poisoning worldwide. Significant differences between substances used in low-income and Western countries confirmed previous literature data. In rural areas and Asian countries, most suicides by poisoning occur mainly through the use of pesticides such as organophosphates and carbamates. In Western countries, illicit drugs (especially opioids) and medically prescribed drugs, such as antidepressants, anxiolytics, and neuroleptics, are the leading cause of suicide by self-poisoning. The selected studies are missing information regarding sociodemographic factors, medical history, and psychopathological factors. Moreover, not all geographic areas are represented in the study and given that the data emerge from autopsy studies, the results could not be representative of the epidemiological scenario. To highlight the involved risk factors and implement a tailored prevention program worldwide, future research should shed light on the correlation between social, medical, and demographic characteristics and the autopsy findings in suicide by self-poisoning. Autopsy and toxicological analyses are crucial to assess the manner of death by suicide and the substances consumed to commit suicide. Performing a complete autopsy on a suspected suicide by self-poisoning could be essential for supporting worldwide public health measures and policy makers; therefore, complete autopsies must be vigorously promoted.

## Figures and Tables

**Table 1:** Summary of the systematic review (ELISA: enzyme-linked immunosorbent assay; EMIT: enzyme-multiplied immunoassay technique; GC: gas chromatography; GC-ECD: gas chromatography-electron capture detection; GC-FID: gas chromatography–flame ionization detection; GC-MS: gas chromatography–mass spectrometry; GC-NP: gas chromatography nitrogen–phosphorus; GC-NPD: gas chromatography-nitrogen phosphorus detection; GS-FID: gas chromatography-flame ionization detection; HGC-FID: headspace gas chromatography-flame ionization detection; ICP-MS: inductively coupled plasma mass spectrometry; LC-MS: liquid chromatography–mass spectrometry; LC-MS-MS: liquid chromatography–tandem mass spectrometry; LLE: liquid-liquid extraction; N-P: nitrogen-phosphorous; SM: spectrophotometric method; UV-VIS: ultraviolet-visible spectrophotometer).

Authors/ Year	Country	Study Design	Sample	Age/ Gender	Cause and Manner of Death	Anamnesis	Biological Matrix/ Toxicological Method (BM/TM)	Toxicological Reports
Rasheed A. [18] 2022	Pakistan	Retrospective (2021)	Suicide autopsy series (*n* = 313)	0–80 M 68 F32	N° of poisoning cases 100/300 (33.33%) Suicide by consuming poison 94/100 (94%) Alleged 4/100 (4%) Accidental 2/100 (2%)	Financial problem Failure in love Ill-health Marital problem Unascertained Problem related to education Family problem Unemployment	Not specified	The most common poison encountered: Organophosphorus 55/100 (**50%**) Phosphide ions 33/100 (**30%**)
Chaudhari V.A. [19] 2022	India (South India)	Retrospective (2007–2017)	Suicide by self-poisoning autopsy series (*n* = 595)	<19 and >60 Mean age 35.81 M 363 F 323	Suicide by consuming poison 595/595 (100%)	Family problem 180 (30.2%) Chronic illness 168 (28.2%) Financial problem 66 (11%) Psychiatric problem 58 (9.7%) Academic failure 42 (7%) Problem in love life 15 (2.5%) Not documented 32 (5.3%)	Not specified	Poisoning agent used in 564/595 cases: Organophosphates 275 (**48.8%**) Plant origin 77 (**12.9%**) Rodenticides 76 (**13.5%**) Unknown 38 (**6.7%**) Organochlorines 32 (**5.7%**) Carbamates 24 (**4.3%**) Miscellaneous 18 (**3.2%**) Hair dye 9 (**1.6%**) Sedatives 7 (**1.2%**) Pyrethroids 5 (**0.9%**) Combination of poisons 3 (**0.5%**)
Collados-Ros A. [20] 2022	Spain	Retrospective (2013–2018)	Suicide autopsy series (*n* = 355)	Not specified	Suicide by consuming poison46/355 (13%) Drug overdose 46/355 (13%) CO poisoning 2/355 (0.6%) Drowning 7/355 (2%) Firearm 26/355 (7.3%) Hanging 176/355 (49.6%) Fall from a height 76/355 (21.4%) Stabbing 8/355 (2.2%) Suffocation 4/355 (1.1%) Traffic accident 10/355 (2.8%)	Not specified	BM: Not specified TM: GC, HGC-FID, spectrometry	Suicide with positive toxicology: Drug overdose in 22.7% of the cases The most common drug classes: Alcohol Benzodiazepines Carbon monoxide Antidepressants Cyanide Benzoylecgonine Cannabinol Paracetamol Morphine
Goswami O. [21] 2021	India	Retrospective (not specified)	Suspected acute poisoning cases (*n* = 584)	0 to >60 M 152 F 92	N° of poisoning cases 244/584 (41.8%) Suicide by consuming poison 210/244 (86%) Accidental poisoning 34/244 (13.9%)	Not specified	BM: Viscera and blood TM: Not specified	Organophosphate 151 (**61.9%**) Carbamates 45 (**18.4%**) Organochlorines **23** (**9.4%**) Nitric acid 17 (**7.0%**) Oxalic acid 8 (**3.3%**)
Cuchara B. [22] 2020	USA	Retrospective (2009–2016)	Suicide autopsy series (*n* = 394)	<12 and >94 Mean age 44.5 M 307 F 87	Consuming poison 71/394 (18%) Hanging 123/394 (31.2%) Firearm 80/394 (20.3%) Blunt impact 58/394 (14.7%) Suffocation 30/394 (7.6%) Sharp force 13/394 (3.3%) Thermal injury 12/394 (3%) Drowning 7/394 (1.7%)	Psychiatric disorders 181 (45.9%)	Not specified	The most common drug classes: Benzodiazepines Opioids Antidepressants Ethanol
Xiao L. [23] 2020	China	Retrospective (2010–2018)	Poisoning autopsy series (*n* = 782)	<1 and >86 Mean age 37 M 511 F 271	Suicide by consuming poison 300/782 (38.4%) Accidental poisoning 391/782 (50%) Homicide by poisoning 39/782 (5%)	Not specified	BM: Blood, urine, stomach content TM: GC, GC-MS, LC-MS, ICP-MS, ELISA	Pesticides 234 (**78%**) Toxic gases 44 (**14.6%**) Medical drugs10 (**3.3%**) Inorganic compounds 8 (**2.6%**) Metal and nonmetal poisons 2 (**0.6%**) Volatile poisons 1 (**0.33%**) Natural plants and naturals 1 (**0.33%)**
Pan M. [24] 2019	China	Retrospective (2017)	Autopsy series (*n* = 299)	Not specified	N° of fatal poisoning cases 39/299 (13%) Self-poisoning 5/39 (12.8%) Accidental poisoning 27/39 (69.2%) Homicide by poisoning 3/39 (7.7%) Undetermined 4/39 (10.3%)	Not specified	BM: Urine, blood, hair, liver TM: GC–FID, LC-MS-MS, G-MS, GC-FID, GC-NPD, GC-ECD, ICP-MS, UV-VIS	Pesticides 3 (**60%**) CO_2_ (**40%**)
Wang Lin-Lin [25] 2019	China	Retrospective (2008–2017)	Autopsy series (*n* = 1968)	<10 and >60 Mean age 35.6 M 78 F 62	N° of poisoning cases140/1968 (7.1%) Suicide by consuming poison 39/140 (27.86%) Accidental poisoning 93/140 (66.43%) Homicide by poisoning 5/140 (3.57%) Undetermined 3/140 (2.14%)	Not specified	BM: Blood, urine, stomach content TM: GC–MS, LC–MS, SM	Drug of addiction 25 (**17.8**) Medical drugs 22 (**15.7%**) Agrochemical poisons 38 (**27.1%**) Respiratory dysfunction toxics 37 (**26.4%**) Poisoning plant/animal 9 (**6.4%**) Alcohol and methanol 5 (**3.6%**) Other chemicals 4 (**2.9%**)
Gharbaouoi M. [26] 2018	Tunisia	Descriptive and crosssectional (2005–2015)	Self-poisoning autopsy series (*n* = 204)	13–86 Mean age 34.2 M 83 F 121	Suicide by consuming poison 204/204 (100%)	History of suicide attempts 42 (20.6%) History of suicide threats 45 (22.1%) Psychiatric disorder 80 (39.2%)	Not specified	Drugs 107/204 (**52.5%**) Pesticides 87/204 (**42.6%**) Caustic product 9/204 (**4.4%**) Butane gas 1 (**0.5%**)
Kordrostami R. [27] 2017	Iran	Retrospective (2011–2015)	Suicide autopsy series (*n* = 1667)	Mean age 32.61 M 462 F 212	Suicide by consuming poison 764/1667 (45.8%)	Not specified	BM: Blood, urine, bile TM: LLE	Pesticides 644 (**84.2%**) Opioids 52 (**6.8%**) Methamphetamine 21 (**2.74%**) Cyanide 17 (**2.2%**) Ethanol 10 (**1.3%**) Strychnine 10 (**1.3%**) Benzodiazepines 6 (**0.8%**) Tricyclic antidepressants 4 (**0.5%**)
Jones A.W. [28] 2016	Sweden	Retrospective (1993–2010)	Poisoning autopsy series (*n* = 13,963)	Not specified	Suicide by consuming poison 4843/13,963 (34.68%) Accidental poisoning 4331/1396 (31%) Undetermined 4789/13,963 (34.3%)	Not specified	BM: Blood TM: EMIT, CG, N–P, GC–NP, GC–MS, LC–MS	The most common drug classes detected in poisoning cases: Ethanol Paracetamol Diazepam Propoxyphene Morphine Propiomazine Zopiclone Citalopram Flunitrazepam Alimemazine
Franchi A. [29] 2016	France	Comparative and retrospective (2002 and 2012)	Suicide autopsy series (*n* = 486)	<14 and >85 Mean age 49.56 M 323 F 163	Poisoning 95/486 (19.5%) Hanging 150/486 (30.86%) Firearm 55/486 (11.3%) Fall from a height 105/486 (21.6%) Other 69/486 (14.19%)	Psychiatric disorders 315 (64.8%)	BM: Blood TM: Not specified	The most common drug classes: Prescription drugs
Azmak A.D. [30] 2015	Turkey	Retrospective (1984–2004)	Suicide autopsy series (*n* = 137)	14–95 M 107 F 30	Poisoning 27/137 (19.7%) Hanging55/137 (40.1%) Firearm 43/137 (31.4%) Drowning 7/137 (5%) Fall from a height4/137 (2.9%) Cutting 1/137 (0.7%)	Not specified	Not specified	Drug overdose 14 (**51.8%**) Pesticides 10 (**37%**) Corrosives 2 (**7.4%**) Methanol 1 (**0.7%**)
Escoferry C.T. [31] 2015	Jamaica	Retrospective (1980–1999)	Poisoning cases series (*n* = 22)	2–69 Mean age 27 M 13 F 9	Suicide by consuming poison 14/22 (63.6%) Accidental poisoning 8/22 (36.4%)	Not specified	Not specified	Paraquat 5 (**35%**) Combination of antipsychotic drugs 2 (**14.2%**) Amitriptyline 1 (**7.1%**) Chlorpropamide 1 (**7.1%**) Alcohol, cocaine 1 (**7.1%**) Paracetamol, chlorpheniramine, ampicillin 1 (**7.1%**) Herbicide 1 (**7.1%**) Toilet bowl cleaner 1 (**7.1%**) Chlorpromazine 1 (**7.1%**)
Delaveris G.J.M. [32] 2014	Norway	Retrospective (2000–2009)	Autopsy series (*n* = 1603)	20–59 Mean age 34.9	Suicide by consuming poison 114/1603 (7.11%) Non-intoxication suicide 119/1603 (7.42%) Accidental intoxication 1204/1603 (75.1%) Homicide 44/1603 (2.74%) Other 122/1603 (7.6%)	Psychiatric problem (not specified)	BM: Blood TM: EMIT, CG	The most common drug classes: Narcotic drugs Benzodiazepines Analgesics Antidepressants Antipsychotics
Dias D. [33] 2013	Portugal	Retrospective (2003–2009)	Suicide autopsy series (*n* = 304)	15 to >75 Not specified	Poisoning 97 (31.9%) Hanging 119 (39.1%) Drowning 22 (7.2%) Firearm 34 (11.2%) Fall from a height 18 (5.9%) Trucidation 11 (3.6%) Other 9 (3.1%)	Not specified	Not specified	The most common drug classes: Ethanol Prescription drugs Illicit drugs Pesticides Others
Vassiliki A.B. [34] 2013	Greece	Retrospective (1998–2010)	Autopsy series (*n* = 1681)	16–97 M 99/126 poisoning cases F 27/126 poisoning cases	N° of poisoning cases126/1681 (7.49%) N° of suicides 257/1681 (1.18%) Suicide by consuming poison 20/257 (16%)	Not specified	BM: Blood, urine TM: GC-MS	Drug of abuse 76 (**60%**) Carbon monoxide 25 (**19.8%**) Pesticides 12 (**9.7%**) Corrosives 6 (**4.8%**) Pharmaceuticals 6 (**4.8%**) Spider bite 1 (**0.8%**)
Frost J. [35] 2012	Norway	Retrospective (2007–2009)	Autopsy series (*n* = 364); numberof cases subjected to toxicological analysis 361/364 (99.1%); number of poisoning cases 74/361 (20.4%)	<30 and >59 M 271 F 93	N° of poisoning cases74/361 (20.4%) Suicide by consuming poison 9/361 (2.5%) Natural death 125/361 (34.6%) Accidental poisoning 65/361 (18%) Road traffic accident 30/361 (8.2%) Accidental fall 12/361 (3.28%) Other accident 52/365 (14.2%) Suicideby another means 59/361 (16.3%) Homicide 9/361 (2.4%)	Not specified	BM: Urine, blood TM: GC, LC-MS	Positive toxicology 8/9: Drugs 4 (**50%**) Alcohol and drugs 3 (**37.5%**) Alcohol 1 (**12.5%**)
Holmgren A. [36] 2010	Sweden	Retrospective (1998–2007)	Suicide autopsy series (*n* = 11,441)	Mean age 51.3 M 8138 F 3303	Poisoning 2462/11,441 (22%) Hanging 4471/1441 (39%) Asphyxia by gas 509 (4.4%) Drowning 803 (7%) Firearm 1307 (11.4%) Fall from a height 632 (5.5%) Sharp-force injury 363 (3.1%) Other 891 (7.8%)	Not specified	BM: Blood, urine, vitreous humor TM: Not specified	The most common drug classes: Paracetamol Dextropropoxyphene Propiomazine Zopiclone Diazepam Citalopram Zolpidem Morphine/codeine Flunitrazepam Alimemazine
Mohanty S. [37] 2007	India	Retrospective (2000–2003)	Suicide autopsy series (*n* = 588)	11 to60 M 294 F 294	Poisoning 180/588 (30.6%) Hanging 192/588 (32.6%) Burns 108/588 (18.3%) Railway 100/588 (17%) Drowning 8/588 (1.3%)	Not specified	Not specified	Organophosphorus128 (**71.1%**) Native plants20 (**11%**) Corrosive acids 16 (**8.8%**) Rodenticides12 (**6.6%**) Phenyl 4 (**2.2%**)
Jonsson A. [38] 2007	Sweden	Retrospective (2002–2003)	Autopsy series (*n* = 10,273) Classified as drug abusers (N° 743/10,273)	Mean age 38	Suicide by consuming poison 97/743 (13%) Accident 293/743 (39.4%) Homicide 20/743 (2.69%) Undetermined 172/743 (23.1%) Natural death 161/743 (21.6%)	Not specified	BM: Blood, urine, vitreous TM: Not specified	No illicit drugs detected 52 (**53.6%**) Illicit drugs detected 44 (**45.3%**) No toxicology performed 1 (**1.03%**)
ShieldsL.B.E. [39] 2006	Kentucky	Retrospective (1993–2002)	Suicide autopsy series (*n* = 2864)	11–96 Mean age 42 M 2340 F 524	Drug toxicity 283/2864 (9.8%) Firearm 1932/2864 (67.4%) Hanging 393/2864 (13.7%) Carbon monoxide intoxication 260/2864 (9%)	Non specified	BM: Blood, urine TM: Not specified	The most common drug classes: Ethanol Antidepressants Benzodiazepines Opioids Cannabinoids Cocaine or metabolites
Jonsson A. [40] 2004	Sweden	Retrospective (1992–2002)	Autopsy series (*n* = 60,313)	0–100 Mean age 46 M 4277 F 2689	N° of poisoning cases6966/60,313 (11.5%) Suicide by consuming poison 3091/6966 (44.3%) Accidental poisoning 581/6966 (8.34%) Undetermined cases by poisoning 3294/6966 (47.2%)	Not specified	BM: Femoral blood TM: Not specified	The most common drug classes: Propoxyphene Amitriptyline Zolpidem Carisoprodol Alprazolam Thioridazine Methadone Ketobemidone
Lahti R.A. [41] 2003	Finland	Retrospective (1997)	Poisoning autopsy series (*n* = 500)	13–92 M 300 F 200	Suicide by consuming poison 325/500 (65%) Accidental poisoning 99/500 (19.8%) Undetermined 76 (15.2%)	Not specified	Not specified	Verified principal drug in 324/325 cases of suicide by poisoning: Neuroleptics 104 (**32%**) Antidepressants 76 (**23.4%**) Opioids 44 (**13.5%**) Hypnotics 33 (**10.1%**) Cardiovascular drugs 29 (**8.9%**) Antidiabetics 11 (**3.3%**) Other drugs 10 (**3%**) Antiepileptics 8 (**2.4%**) Anxiolytics 5 (**1.54%**) Other analgesics **3** (**0.9%**) Amphetamines **1** (**0.3%**)

## Data Availability

Data sharing is not applicable; no new data were created or analyzed in this study.
